# Utilisation of the web-based Home Assessment Tool among patients with COVID-19 in Selangor, Malaysia: An observational study

**DOI:** 10.51866/oa.205

**Published:** 2024-03-16

**Authors:** Samat Farhani, Abdul Jalil Roslina, Mohammad Nik Mazlina, Hassan Noor Hasliza, Lau Lih Bing, Sulaiman Noorul Amilin, Zainol Rashid Zienna Zufida, Rosnan Siti Khalimah

**Affiliations:** 1 MD, MMed (FamMed), Family Medicine Specialist, Tanjung Karang Health Clinic, Kuala Selangor, Selangor, Malaysia. Email: hani5204@gmail.com; 2 MBBS, MFamMed, Family Medicine Specialist, Klinik Kesihatan Sekinchan, Sabak, Bernam, Selangor, Malaysia.; 3 MBBS, MMed (FamMed), Family Medicine Specialist, Klinik Kesihatan Kelana Jaya, Selangor, Malaysia.; 4 MD, MMed (FamMed), Family Medicine Specialist, Klinik Kesihatan Sungai Pelek, Selangor, Malaysia.; 5 MD, FRACGP, Family Medicine Specialist, Klinik Kesihatan Batu 8, Gombak, Selangor, Malaysia.; 6 MBBBS, MFamMed, Family Medicine Specialist, Klinik Kesihatan Gombak Setia, Selangor, Malaysia.; 7 MBBS, MFamMed, Family Medicine Specialist, Klinik Kesihatan Rawang, Selangor, Malaysia.; 8 MBBS, Medical officer, Klinik Kesihatan Sungai Tengi Kanan, Kuala Selangor, Selangor, Malaysia.

**Keywords:** COVID-19, Web-based intervention, Home isolation

## Abstract

**Introduction:**

The COVID-19 pandemic has caused many countries to turn to web-based solutions. The Home Assessment Tool (HAT) is a web-based system using the MySejahtera application developed by the government. It serves as a communication platform for patients with COVID-19 to self-monitor their clinical symptoms and enables authorised healthcare personnel to access and manage collected data for clinical monitoring. Our study aimed to examine the utilisation of this internet-based tool among patients with COVID-19 in Selangor.

**Methods:**

This observational study analysed secondary data from the self-reported HAT within MySejahtera. It included all patients who were diagnosed with COVID-19 through molecular assays such as RT-PCR or RTK-Ag on 1–21 February 2021, aged >18 years and residing in Selangor. Patients who had documented their symptoms at least once in the HAT during the prescribed 10-day isolation period were classified as HAT users.

**Results:**

A total of 4438 patients were included, of whom 39.4% were HAT users, while 60.6% were non-HAT users. Logistic regression analysis revealed three significant factors associated with low utilisation of the HAT: absence of medical condition (odds ratio [OR]: 9.4; 95% confidence interval [CI]: 7.49–12.01), advanced age (OR: 1.35; 95% CI: 1.20–1.52) and non-Malaysian citizenship (OR: 3.4; 95% CI: 2.50-4.72).

**Conclusion:**

The utilisation of the HAT is low, which is associated with advanced age (>65 years), absence of medical conditions and foreign nationality. It is imperative to develop inventive strategies tailored to address the unique needs of these particular demographics.

## Introduction

The COVID-19 pandemic, which originated in Wuhan, China, has had significant ramifications worldwide. According to Elengoe,^1^ the initial case of COVID-19 in Malaysia was detected on 25 January 2020, subsequent to direct interaction with an individual who had contracted the virus in Singapore. According to Jayaraj et al.,^2^ there was a notable rise in the average incidence rate over a 14-day period, which escalated from two cases per 100,000 patients from March to May 2020 to 55 cases per 100,000 patients from December 2020 to February 2021. The central region, comprising Selangor and Kuala Lumpur, consistently exhibited higher incidence rates than did the eastern region. The central region experienced an incidence rate of 104 cases per 100,000 patients from December 2020 to February 2021.^2^

COVID-19 represents a range of diseases wherein the condition of patients may deteriorate progressively. Consequently, it is imperative to diligently observe patients for a number of days during their isolation period to detect red-flag symptoms such as chest pain, respiratory distress and fatigue. In the early stages of the COVID-19 pandemic in 2020, healthcare professionals in Selangor employed diverse monitoring mechanisms, such as telephone communication and self-reporting via Google Sheets, to evaluate the symptoms of patients in their residences. Nevertheless, these practices lacked standardisation and were contingent upon localised agreements.

The Home Assessment Tool (HAT) was initially implemented in January 2021 through the MySejahtera application.^3,4^ The primary objective of this development was to alleviate the strain on primary care clinics caused by the need to evaluate a substantial volume of patients with COVID-19. Currently, the implementation of web-based HAT monitoring enables patients with COVID-19 to engage in self-evaluation of their symptoms and effectively communicate this information to the application.^4^ The instrument facilitates expedited access for authorised healthcare professionals to patients’ demographic details, geographical location, medical condition and self-reported symptoms and severity related to COVID-19. Patients who voluntarily disclose any indication of potential health issues are contacted via telephone to conduct a more comprehensive assessment.^4^

In various countries that have encountered similar difficulties, there has been an extensive utilisation of web-based application solutions amidst the pandemic.^5,6^ Healthcare providers in the United Kingdom have implemented video or telephone consultations as a means of mitigating the psychological burden associated with hospital visits for patients diagnosed with COVID-19.^7^ The Centers for Disease Control and Prevention developed a self-assessment tool for patients diagnosed with COVID-19, with the aim of facilitating community empowerment in symptom evaluation and counselling.^8^ In the study by Mehring et al.,^5^ the utilisation of a web-based tool was found to yield a recording rate of 69.6% for outcomes based on a sample size of 300,000 digital assessments. Among these recorded outcomes, a significant majority (80.5%) indicated the presence of mild symptoms associated with COVID-19. The study was limited by its scope, precluding the authors from making a recommendation regarding the instrument.

Digital technology has played a significant role in the management of COVID-19 in various countries.^6,9,10^ For example, Iceland has employed mobile technology to oversee self-reported symptoms and South Korea and Singapore to identify and monitor contacts. China, Australia, Taiwan, South Korea and Hong Kong have utilised a diverse range of technological tools to monitor the adherence to quarantine measures and identify instances of non-compliance among patients infected with COVID-19 placed under home quarantine.^6^ According to Whitelaw et al.,^6^ virtual care platforms and remote monitoring are employed in Canada, the United States and Australia for the treatment of COVID-19.

Despite the abovementioned context, there has been a scarcity of research pertaining to the utilisation of the HAT in Malaysia. Thus, our study aimed to examine the utilisation of the web-based HAT during the early stages of its integration among patients with COVID-19 in Selangor.

## Methods

This observational study analysed secondary data from the HAT integrated within MySejahtera. It included all patients who were diagnosed with COVID-19 through molecular assays, specifically Reverse Transcriptase Polymerase Chain Reaction (RT-PCR) or Antigen Rapid Test kit (RTK-Ag), aged at least 18 years and residing in the state of Selangor. These patients had their personal information entered and integrated into MySejahtera from the laboratory. All patient data were collected from MySejahtera on 1–21 February 2021.

### Research instruments

In MySejahtera, patients receive notifications to complete the self-reported HAT on a daily basis for 10 days during the isolation period regardless of whether they are experiencing symptoms. They have the option to utilise a mobile device or any computerised device with internet connectivity to complete the questionnaire. In this study, data on the demographic characteristics of patients, along with a comprehensive list of general symptoms associated with COVID-19 that had been reported, such as fever, nausea, vomiting, sore throat, diarrhoea, cough, anosmia and ageusia, were collected. Additionally, the presence of red-flag symptoms, including difficulty of breathing, chest pain, blue lips and feelings of weakness or drowsiness, was documented.^11^ Patients who had documented their symptoms at least once in the HAT during the prescribed 10-day isolation period were classified as HAT users.

### Statistical analysis

IBM SPSS version 26 (IBM, Chicago, IL) was used for the data analysis. Categorical variables were presented as frequencies and percentages. Pearson’s chi-square test was used to determine the association between usage of the HAT and demographic characteristics of the sample. Logistic regression analyses were performed to measure the odds ratios (ORs) of HAT usage among the sample.

## Results

A total of 4438 patients diagnosed with COVID-19 were reported from the laboratory to MySejahtera within 20 days of the study period. However, only 39.4% (n=1749) of the patients were identified as HAT users, while 60.6% were non-HAT users. The proportion of the male non-users was almost equal to that of the female users. The mean age of the HAT users was 35.6 (SD: ±12) years. The older adults (aged ≥65 years) showed less use of the HAT. Many patients who resided in Petaling, Hulu Langat and Klang used the HAT more than did the patients who resided in other districts in Selangor. Only 16.8% (n=48/238) of the non-Malaysian citizens used the HAT during the home isolation period. The associated factors among the demographic data are presented in Table 1. The analysis showed that advanced aged (≥65 years), absence of a medical condition and non-Malaysian citizenship were significantly associated with low utilisation of the HAT.

**Table 1:** Demographic data and their association with home assessment tool (HAT) usage among the patients with COVID-19.

Variables	HAT users n (%)	Non-HAT users n (%)	Exp (B)	95% CI	P-value
**Sex** Male Female	930 (38.3) 819 (40.8)	1500 (61.7) 1189 (59.2)	0.9	0.78-1.01	0.08
**Age, year** 18-39 40-64 ≥65	1240 (41.6) 492 (36.0) 17 (18.5)	1740 (58.4) 874 (64.0) 75 (81.5)	1.35	1.20-1.52	0.000
Mean age (SD), year	35.6 (±12)		1.01	1.00-1.01	0.000
**Background medical condition** Yes No	245 (56.3) 1299 (54.4)	190 (43.7) 1009 (45.6)	9.4	7.49-12.01	0.000
**District** Gombak Klang Kuala Langat Kuala Selangor Petaling Sabak Bernam Sepang Hulu Langat Hulu Selangor	157 (42.4) 260 (53.7) 42 (42.0) 50 (37.9) 633 (39.1) 7 (29.2) 62 (39.7) 427 (40.1) 111 (38.4)	213 (57.6) 424 (46.3) 58 (58.0) 82 (62.1) 984 (60.9) 17 (70.8) 94 (60.3) 639 (59.9) 178 (61.6)			0.982
**Nationality** Malaysian Non-Malaysian	1701 (41.0) 48 (16.8)	2451 (59.0) 238 (83.2)	3.4	2.50-4.72	0.000

In total, 183 (10.46%) patients had reported red-flag symptoms, mainly difficulty of breathing and chest pain. There was no association found between the demographic factors and the presence of red-flag symptoms among the HAT users (Table 2).

**Table 2:** Association between the presence of red-flag symptoms and demographic factors among the HAT users.

Variables	With red-flag symptoms, n (%)	Without red-flag symptoms, n (%)	P-value
**Age, year**			
18-39	141 (11.4)	1099 (88.6)	0.137
40-64	40 (8.1)	452 (91.9)	
≥65	2 (11.8)	15 (88.2)	
**Sex**			
Male	87 (9.4)	843 (90.6)	0.1
Female	96 (11.7)	723 (88.3)	
**Background medical condition**			
Yes	28 (11.4)	217 (88.6)	0.78
No	132 (10.2)	167 (89.8)	
**Nationality**			
Malaysian	179 (10.5)	1522 (89.5)	0.625
Non-Malaysian	183 (10.5)	1566 (89.5)	

**Figure 1:**
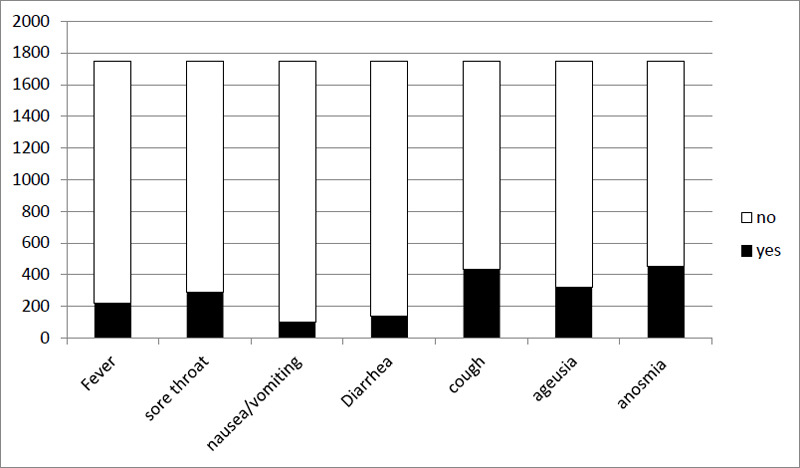
Distribution of the general symptoms among the HAT users.

Figure 1 shows the distribution of the general symptoms among the HAT users. In the tool, patients can report more than one symptom, and the symptoms among all HAT users are accumulated during their 10-day isolation period. The diagram depicts that cough, anosmia, ageusia and sore throat remain the most common symptoms reported by patients in the HAT.

## Discussion

The findings of our study illustrate the application of MySejahtera as a means to offer patients with COVID-19 self-educational resources and a symptom monitoring diary. The adoption rate of the HAT through MySejahtera is relatively lower in this study than in previous studies.^12^ In their study, Timmers et al.,^12^ revealed that 86% of patients were able to utilise mobile applications substantially well during the COVID-19 pandemic and adhere to self-health assessment reports as required. The average age of their users was 50 years, in contrast to the present findings. Medina et al.,^13^ reported an almost similar level of engagement between patients and applications to that in the study by Timmers et al. However, they did not explore any significant factors that influenced this engagement.

The present study examined several factors associated with low utilisation of the HAT. These factors included the absence of medical conditions, advanced age and foreign nationality. Timmers et al.,^12^ also found that patients in the older age group (65 years and older) exhibited a lower propensity to utilise the web application throughout their study, in contrast to other reports. The non-Malaysian citizens also demonstrated limited utilisation of the application. Given their heightened susceptibility to experiencing severe manifestations of COVID-19, the patients with a medical condition who had contracted the virus displayed a growing inclination towards utilising the internet-based HAT during their period of self-isolation at home. Based on these findings, it is advisable to encourage the usage of the HAT among patients aged 65 years and above and non-Malaysian citizens to promote wider adoption. Many factors may contribute to these findings, such as limited proficiency in information technology, linguistic challenges and insufficient assistance from family members or caregivers. According to Walton et al.,^9^ patients of different profiles necessitate different levels of remote monitoring support.

### Significance

The present study presents an overview of the utilisation of the web-based HAT since its introduction in January 2021. The outcomes of this study can aid relevant authorities in identifying effective strategies to enhance the utilisation of a web-based self-reporting application among patients infected with COVID-19. Additionally, the study provides data on the factors associated with usage of the HAT, particularly among individuals who are more likely to not use the tool.

### Limitations

As this study used secondary data from the HAT through MySejahtera, the data analysis was limited. The factors identified in this study did not yield true Odd Ratios; thus, further studies such as case-control studies are needed to determine associated factors. This study was also limited to a specific region in Malaysia and was conducted over a period of 3 weeks. Furthermore, during the study, MySejahtera was identified as one of the limited number of web-based or remote solutions accessible. Consequently, patients lacking MySejahtera identification were not included in the enrolment process. Patients admitted to the hospital and diagnosed with COVID-19 may encounter challenges in providing selfreported information through the designated application. Therefore, it is not possible to extrapolate the results of the study to the entire population of the country.

### Recommendations

The consideration of expanding remote monitoring options using MySejahtera is warranted. The utilisation of telephone follow-ups is recognised as a valuable and economical strategy, complemented by the incorporation of video calls in cases where an escalation of care is necessary.^14,15^ Additionally, the use of wearable sensors in high-risk, vulnerable populations for COVID-19 is recommended to allow for timely identification of disease progression.^16^

Several suggested interventions that have the potential to enhance the adoption of the web-based application among non-users encompass integrating the application into an established workflow and adapting the application into a prototype that is specifically tailored for older patients. A prototype featuring an expanded range of multilingual options would effectively cater to the diverse linguistic needs of Malaysia’s multiethnic population as well as patients from non-Malaysian backgrounds. During the enrolment period, the implementation of application enhancements that enable the automated sending of regular reminders through text messaging has the potential to enhance patient engagement.

Finally, it is recommended that further research be undertaken, encompassing a broader range of Malaysian states and employing larger sample sizes, to achieve a more comprehensive representation of the Malaysian population. Following the introduction of the HAT and the implementation of targeted interventions, it is advisable to undertake further research after a certain period has passed.

## Conclusion

The utilisation of the HAT among patients with COVID-19 is low. Advanced age (65 years and older), absence of medical conditions and non-Malaysian citizenship are associated with such low utilisation. It is imperative to develop inventive solutions tailored to these findings to improve the utilisation of the HAT.

## Data sharing statement

The data of the study are available from the corresponding author and can be provided upon request.

**How does this paper make a difference in general practice?**

- The study provides an overview of the utilization of the web-based Home Assessment Tool (HAT) in Malaysia.
- Policy makers and clinicians should prioritize addressing the low utilization rates of the HAT and its contributing factors.
- Improving the application and implementing a systematic technical remote monitoring for patients with COVID-19 during home quarantine is essential
